# Intravenous occlusive lipoma: Case report and literature review

**DOI:** 10.1016/j.xjtc.2026.102316

**Published:** 2026-03-14

**Authors:** Silvano Marcello, Secer Rukiye, Bohnenberger Hanibal, Roy Terrance, Hinterthaner Marc, Grambow Eberhard, Elger Florian

**Affiliations:** a Department of Cardiac, Thoracic and Vascular Surgery, University Medical Center Göttingen, Göttingen, Germany; b Division of Vascular and Endovascular Surgery, Department of Cardiac, Thoracic, Vascular Sciences and Public Health, University of Padua, Padua, Italy; c Institute for Pathology, University Medical Center Göttingen, Göttingen, Germany; d Department of General, Visceral, Thoracic, Vascular and Transplantation Surgery, University Medical Centre Rostock, Mecklenburg, Germany

**Figure:**
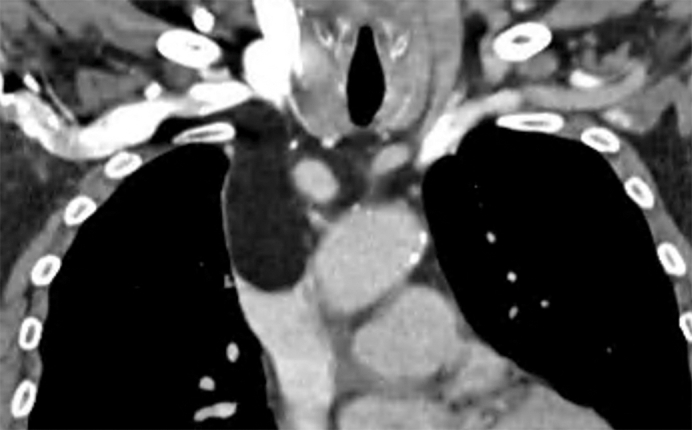
A voluminous hypodense mass fills the lumen of the superior vena cava.

**Central Message**

We report a surgically resected benign SVC lipoma. Surgical removal of intravenous lipomatous tumors may prevent symptom progression and allow definitive diagnosis, even if asymptomatic.

Intravascular lipidic tumors are a rare occurrence, with fewer than 50 cases documented in the literature. Their clinical impact, however, can be significant. Depending on the location of the tumor, patients may present with deep venous thrombosis, cardiac failure, or superior vena cava (SVC) syndrome. Moreover, open surgical treatment for the removal of these masses can be extremely challenging, particularly when the vena cava or brachiocephalic arteries are involved. We report a case of complex surgical treatment of a symptomatic lipoma located in the SVC in a 65-year-old woman with morbid obesity. A literature review also was conducted in regard to this topic.

## Case Report

### Patient Presentation

The patient was a 65-year-old woman who initially presented with a persistent dry cough that has lasted for several months. More recently, her condition had worsened, with the onset of progressive dyspnea (Medical Research Council scale 4) and bilateral swelling of the arms and face.

She had morbid obesity (body mass index 47.4) with a medical history of bronchial asthma and arterial hypertension. Her anamnesis also included cervical cancer treated 38 years earlier with hysterectomy, radiotherapy, and chemotherapy, as well as sarcoidosis diagnosed 15 years earlier, which had completely remitted. She had quit smoking 10 years before presentation.

**Figure 1:**
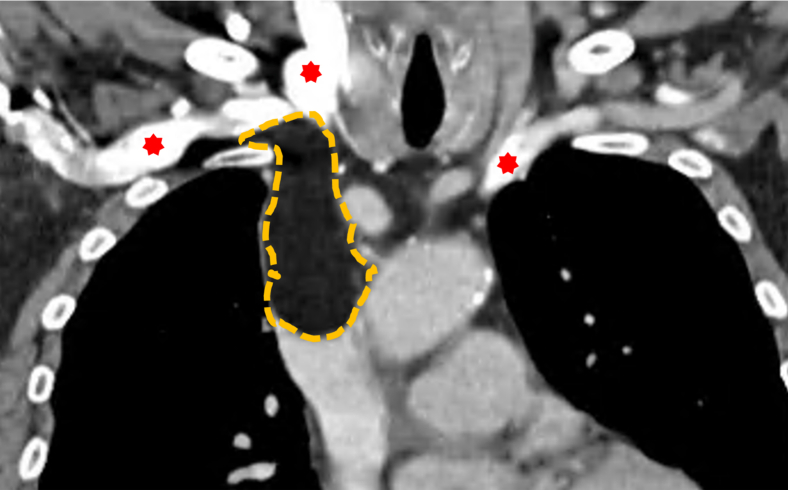
Preoperative computed tomography in venous phase. A voluminous hypodense mass fills the lumen of the superior vena cava in its cranial extent up to the brachiocephalic vein confluence. The *yellow dotted line* shows the edges of the mass. *Red points* mark the stasis of contrast agent in the supracaval veins.

A triple-contrast computed tomography of the chest (Figure 1) revealed a low-density, 8-cm intraluminal mass extending caudally within the SVC, originating from the brachiocephalic junction. The caval lumen was completely occluded, and this finding was confirmed by magnetic resonance angiography. A presumptive diagnosis of vascular sarcoma was made. After multidisciplinary discussion with vascular, thoracic, and cardiac surgeons, surgical resection of the mass was planned. The patient agreed to the procedure, and informed consent for publication was obtained. Institutional review board approval was not required.

### Procedure

Surgical access was obtained under general anesthesia using a modified Dartevelle thoracotomy, distally extended with a sternotomy. After meticulous mobilization of the right lung, which had been temporarily disconnected from ventilation, exposure of the SVC up to the right atrium and of both brachiocephalic veins was achieved. The tumor was clearly visible through the distended venous wall. Two venous cannulas were inserted to decompress the cerebral venous drainage supracavally and to allow reinfusion via the inferior vena cava.

The first cannula, inserted via the left internal jugular vein, was positioned under direct vision in an optimal location; the second cannula was positioned percutaneously in the right femoral vein. After full heparinization and establishment of venous-venous bypass for cerebral and upper thoracic venous drainage, the SVC was clamped above the right atrium, as well as the left brachiocephalic vein caudal to the drainage cannula. A heart-lung machine without an oxygenator was used to run the bypass. The SVC was then opened at the site of maximal tumor expansion. The lipomatous tumor was carefully mobilized from the lumen of the SVC using a broad dissector and gentle digital maneuvers. In the region of the right brachiocephalic vein, tumor mobilization was more demanding because of strong adherence of the tumor to the vessel wall, but successful en bloc removal was achieved.

**Figure 2:**
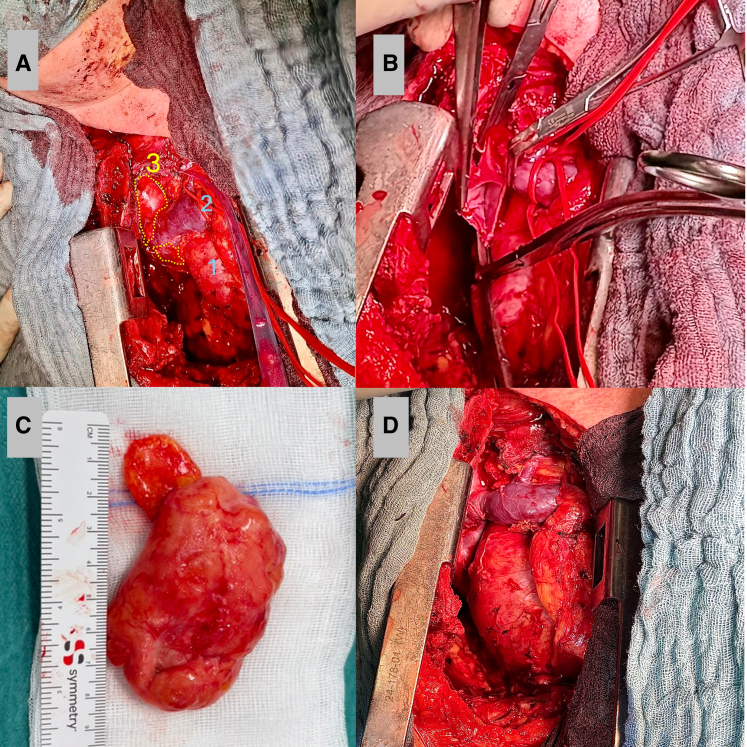
Operative outlines. A, The aorta (1) is visible under the left brachiocephalic vein (2), secured with a red vessel loop. The tumor (3) can be seen through the wall of the superior vena cava (outlined by a *yellow dotted line*) and determines a clear occlusion of the proximal venous axes above can be recognized. B, Venotomy completed and intraluminal mass excised. C, Resected mass of almost 9 × 5 cm. D, Postoperative reconstruction of the venous structures.

After complete mobilization and removal of the intact tumor, the SVC was reconstructed by direct suture after deairing and flushing maneuvers. Blood flow was then restored after a final flush and deairing procedure. Hemostasis was secured, drains were placed, and closure of the thoracotomy/sternotomy and the wound was performed in the standard fashion. The venous cannulas were removed without difficulty, and the puncture sites were compressed for 20 minutes and dressed with a pressure bandage (Figure 2).

### Postoperative Outcomes

The patient was extubated uneventfully in the immediate postoperative period; intermittent noninvasive ventilation with continuous positive airway pressure (inspired oxygen fraction 45%) was required because of impaired chest-wall mechanics related to the presence of severe obesity. Initially, reduced right diaphragmatic motion was observed in the setting of a concomitant pleural effusion, but pulmonary function and ventilatory mechanics improved markedly over time, and no new neurologic deficits were observed. Postoperatively, a reduction in hemoglobin levels (from 13.5 g/dL preoperative to 6.7 g/dL) and bleeding from pleural drainage were noted and effectively managed with transfusion of 2 units of concentrated red blood cells. Amine support was progressively tapered during the first postoperative days.

**Figure 3:**
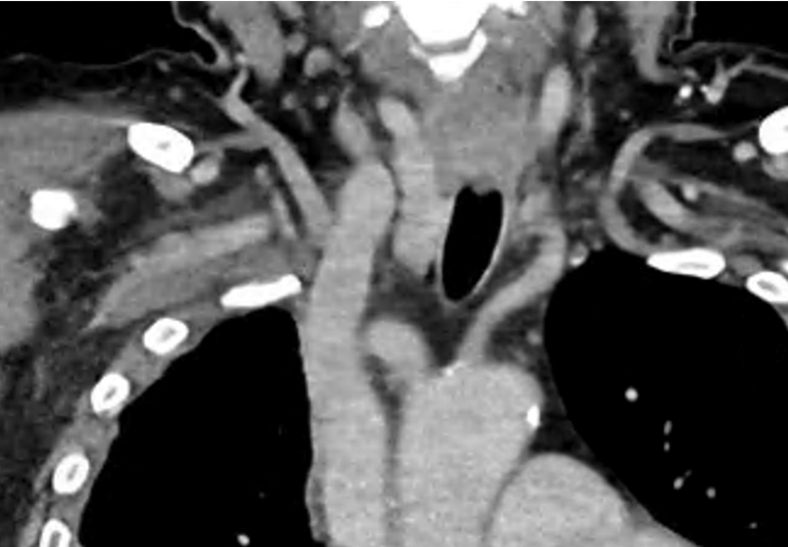
Follow-up computed tomography postoperatively. No residual tumor is visible.

The patient received anticoagulation with high-molecular-weight heparin and diuretic therapy (furosemide 60 mg/day in divided doses). No complications occurred. She was transferred from the intensive care unit to the general ward on postoperative day 2 and was subsequently discharged to a rehabilitation facility on postoperative day 8. A follow-up computed tomography showed a complete mass excision and good patency of the SVC (Figure 3).

### Pathology Findings

**Figure 4:**
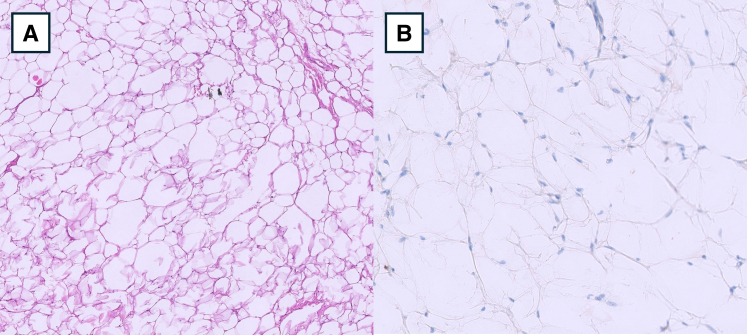
Histologic analysis. A, Detail of lipoma tissue: no cellular atypia, pleomorphism, lipoblasts, or necrosis are detectable. B, MDM2 staining, showing completely negative nuclear expression.

Histologic examination of the resected mass revealed a well-circumscribed, encapsulated lesion composed of mature, uniform adipocytes without evidence of cellular atypia, pleomorphism, lipoblasts, or necrosis. The fibrous capsule was intact, and no infiltration of adjacent structures was observed. There were no histopathologic criteria suggestive of malignancy or features compatible with a well-differentiated liposarcoma. To further exclude an atypical lipomatous tumor or liposarcoma, immunohistochemical staining for MDM2 was performed and showed completely negative nuclear expression, supporting the diagnosis of a benign lipoma (Figure 4).

## Discussion

Intravascular tumors are a rare occurrence and include malignant sarcomas (ie, angiosarcoma) and benign masses. Among these, lipomas appeared to be prominent and have been described in multiple venous districts, including the chest and neck (SVC, brachiocephalic and jugular veins),^1^, ^2^, ^3^, ^4^, ^5^^,^E1, E2, E3, E4, E5, E6, E7, E8, E9, E10, E11, E12, E13, E14, E15, E16, E17, E18 visceral veins (inferior vena cava, renal veins),^E19^^,^^E20^ and lower-limbs veins (iliac and femoral veins).^E21^^,^^E22^ These lesions often determine compressive or occlusive manifestations and enter in the differential diagnosis with angiosarcoma and leiomyosarcoma.^1^ The final diagnosis must be histopathologic, because intravascular infiltration from benign lipomas has been reported.^E17^^,^^E18^ However, radiologic findings of nonadipose materials within the mass may help to discriminate malignant masses.^E24^

In our case, given the extent of the tumor, an open approach was selected. To ensure full exposure of the brachiocephalic vein if required, a Dartevelle thoracotomy was performed and extended caudally with a median sternotomy. In the setting of severe obesity, this provided the necessary operative field; a less-invasive approach was considered unlikely to achieve adequate exposure. Moreover, preoperatively it was not fully clear whether the lesion represented a malignant tumor; therefore, in the worst-case scenario, partial venous resection with subsequent reconstruction might have been required. Venous-venous bypass was established using a cardiopulmonary bypass circuit without an oxygenator, representing a cost-effective alternative to venovenous extracorporeal membrane oxygenation in our setting, which would also have been feasible. Bypass enabled safe clamping of the vena cava above the right atrium and the supracaval veins, facilitating tumor extirpation with reduced venous blood loss, allowing sufficient time for caval reconstruction, and preventing further aggravation of upper venous congestion, thereby improving cerebral venous drainage. This ability to operate without marked time pressure was particularly relevant because the lipoma was highly adherent to the vessel wall at the brachiocephalic vein, and dissection proved more time-consuming than anticipated on the basis of intraoperative macroscopic assessment.

A literature review was performed on PubMed in which we identified 23 cases of lipomas involving the superior vena cava and/or the brachiocephalic vein (Table 1). The superior vena cava was involved in 17 cases and the brachiocephalic vein alone in 15 cases, of which 6 did not involve the SVC. Interestingly, a tumor extension to the right atrium was seen in 9 cases and in 2 cases extravascular extension was present. However, the disease appeared to be an incidental finding in 13 cases. In symptomatic cases, SVC syndrome represents the most common presentation, in 6 cases. Surgical resection was performed in 18 cases, with no recurrences at follow-up after exeresis.

**Table 1:** Collection of the available cases of intravascular lipoma of the SCV and/or BCV

Case	Age, sex	Tumor location	Right atrium involvement	Clinical presentation	Treatment
Vinnicombe, 1994^E16^	42, F	SVC, BCV	Yes	SVC syndrome	Surgery
Thorogood and Maskell, 1996^E15^	73, M	SVC, BCV	No	Incidental finding	Medical follow-up
Trabut, 1999^E14^	55, M	SVC, BCV	No	Incidental finding	Surgery
Moore, 2008^3^	58, M	BCV	No	Incidental finding	Medical follow-up
Mordant, 2010^E13^	55, F	SVC, BCV	Yes	Incidental finding	Surgery
Yoon, 2012^E11^	39, F	BCV, IJV	No	Incidental finding	Surgery
Bravi, 2012^E12^	63, M	SVC, BCV	Yes	Incidental finding	Surgery
Cheezum, 2013^E10^	84, F	SVC	Yes	SVC syndrome	Surgery
Lococo, 2013^E18^	61, M	BCV	No	Incidental finding	Surgery
Tanyeli, 2015^E9^	48, M	SVC	No	SVC syndrome	Surgery
Concatto, 2015^E8^	58, M	SVC	No	Incidental finding	Surgery
Iqbal, 2017^1^	51, M	BCV, IJV	No	Incidental finding	Not reported
Wahab, 2017^E7^	70, F	SVC	Yes	Incidental finding	Surgery
Elen, 2019^E5^	54, M	SVC	Yes	Cardiac arrythmia	Surgery
Beliaev, 2020^E6^	49, F	SVC, BCV	No	Incidental finding	Surgery
Podobed, 2021^E2^	54, F	SVC, BCV	No	SVC syndrome	Surgery
Nasr, 2021^E3^	56, F	SVC	Yes	Cardiac arrythmia	Surgery
Soetisna, 2022^E1^	54, M	SVC	Yes	Cardiac arrythmia	Surgery
Cohen-Mussali, 2023^5^	64, F	SVC, BCV, IJV	No	Incidental finding	Surgery
Sundaram, 2021^E4^	58, M	SVC, BCV, IJV	No	SVC syndrome	Surgery
Roisin, 2024^2^	51, M	BCV	No	SVC syndrome	Medical follow-up
Hiregouja Eranna, 2024^E17^	64, F	BCV	No	Persistent cough	Medical follow-up
Yan, 2025^4^	61, M	SVC	Yes	Incidental finding	Surgery
Present case	65, F	SVC	No	SVC syndrome, Persistent cough	Surgery

*SVC*, Superior vena cava; *BCV*, brachiocephalic vein; *F*, female; *M*, male; *IJV*, internal jugular vein.

Specifically, lipomas localized in the superior vena cava and brachiocephalic vein grow extensively,^E1^^,^^E5^^,^^E16^ remaining asymptomatic for long periods and possibly reaching the right atrium.^4^ These lesions represent a significant surgical challenge owing to the requirement for an open thoracic approach, extracorporeal circulation (cardiopulmonary bypass), and their occasionally considerable extent. Combination of thoracotomy and cervicotomy is required in case of tumor extension in the subclavian or internal jugular veins.^E11^^,^^E13^ In some cases, extracorporeal circulation was spared for brachiocephalic venotomy^5^ or thoracoscopic approaches were used.^4^

Postoperative complications are frequently reported after open chest venotomies and may include pleural effusion, phrenic nerve injury with resultant diaphragmatic paresis, hemothorax, Dressler syndrome,^5^ or pulmonary embolism.^E9^ However, in most cases surgery is curative, without recurrences.

The necessity of operating on asymptomatic patients is still debated. However, given the reported risk of growth,^E24^ thrombosis,^E12^ cardiac failure,^E10^ and arrythmias^E1^^,^^E3^^,^^E5^ in case of heart involvement, surgical management in patients who are fit for surgery may be preferable. Even if the surgical approach may be challenging and invasive (as in the present case), strategies as venous-venous bypass may reduce the risk of stroke, without the necessity of a more complex cardiopulmonary bypass. In addition, the final confirmation of the a benign tumor can only be obtained histologically on a surgically resected specimen, because radiologic findings may be misleading.^E23^ This represents an important issue, given the necessity to apply a specific medical oncologic protocol in case of malignancy, especially in patients who tend to present at a young age (average 58.8 years old).

## Conclusions

Intravascular lipoma of the superior vena cava represents a rare, but underhanded occurrence. The definitive diagnosis can only be determined histologically, and resection may be recommended, even if procedurally challenging and burden potential morbidity.

## Conflict of Interest Statement

The authors reported no conflicts of interest.

The *Journal* policy requires editors and reviewers to disclose conflicts of interest and to decline handling or reviewing manuscripts for which they may have a conflict of interest. The editors and reviewers of this article have no conflicts of interest.
